# Expression of interleukin-8 and integrin β3 predicts prognosis of patients with hepatocellular carcinoma after hepatectomy

**DOI:** 10.1097/MD.0000000000039458

**Published:** 2024-10-11

**Authors:** Jiao Zhang, Yi Yin, Jiliang Tang, Mingze Ma, Huimin Shen, Yingrong Zhang, Fengkai Sun

**Affiliations:** a Department of Gastroenterology, Shandong Provincial Hospital Affiliated to Shandong First Medical University, Jinan, China; b Department of Gastroenterology, Shandong Provincial Hospital, Cheeloo College of Medicine, Shandong University, Jinan, China; c Department of Paediatrics, Shandong Provincial Hospital Affiliated to Shandong First Medical University, Jinan, China; d Department of Paediatrics, Shandong Provincial Hospital, Cheeloo College of Medicine, Shandong University, Jinan, China; e Emergency Department, Rizhao Central Hospital, Rizhao, China; f Department of Infectious Diseases, Shandong Provincial Hospital Affiliated to Shandong First Medical University, Jinan, China; g Department of Infectious Diseases, Shandong Provincial Hospital, Cheeloo College of Medicine, Shandong University, Jinan, China; h Postdoctoral Research Station, College of Acupuncture and Massage, Shandong University of Traditional Chinese Medicine, Jinan, China.

**Keywords:** hepatocellular carcinoma, integrin β3, interleukin-8, prognosis

## Abstract

As important components in the tumor microenvironment, interleukin-8 (IL-8) and integrin β3 play a key role in the progression and metastasis of hepatocellular carcinoma (HCC). This study aimed to determine the expression of IL-8 and integrin β3 and their prognostic value in patients with HCC after hepatectomy. We investigated the expression of IL-8 and integrin β3, their clinical significance, as well as their correlation in the cancer tissue of 130 patients with HCC using immunohistochemistry. The prognostic value of IL-8 and integrin β3 was investigated through the follow-up of patients with HCC after hepatectomy. In HCC, IL-8 expression had a positive correlation with integrin β3 expression. Increased expressions of IL-8 and integrin β3 were indicators of tumor progression and poor prognosis in patients with HCC after hepatectomy. IL-8 positive specimens exhibited a higher proportion of macrovascular invasion, larger tumor size, poor differentiation, and advanced tumor-node-metastasis (TNM) stage (*P* < .05, respectively). Integrin β3 positive group exhibited a higher proportion of TNM III-staged tumors (*P* < .05). The results indicated that macrovascular invasion, advanced TNM stage, and integrin β3 expression were independent unfavorable prognostic factors in HCC after hepatectomy. Integrin β3 expression was proved to be an independent unfavorable prognostic factor in HCC after hepatectomy. Targeting integrin β3 might be a potential therapeutic approach in preventing tumor progression in HCC.

## 1. Introduction

Hepatocellular carcinoma (HCC) ranks the sixth common cancer and the fourth leading cause of cancer-related deaths worldwide.^[1]^ Despite the availability of diverse therapies such as radiofrequency ablation, immunotherapy, and molecular targeted therapy, tumor resection surgery remains the primary therapeutic option for patients with HCC.^[2]^ There is increasing evidence that HCC accumulates mutations that stimulate the progression and exacerbate the outcome of the disease through the tumor microenvironment, which comprises tumor cells, stromal cells, cellular interaction proteins, and cytokines.^[3]^ Therefore, a better understanding of the molecular mechanisms involved in the tumor microenvironment of HCC is essential to accurately predict the prognosis of patients and investigate new therapeutic strategies.

The tumor microenvironment is the basis for cancer progression and metastasis. Pro-inflammatory cytokines produced in the tumor microenvironment play a critical role in cancer-related inflammation, invasion, and metastasis.^[4]^ Interleukin-8 (IL-8), secreted by both tumor cells and inflammatory cells, is identified as a key regulator in this process.^[5,6]^ IL-8 promotes tumor angiogenesis and metastasis through binding to high-affinity cell surface receptors CXC chemokine receptor 1 and CXC chemokine receptor 2.^[6,7]^ Over-expression of IL-8 has been detected in variety of tumors and reported to play a crucial role in carcinogenesis, angiogenesis, invasion, and metastasis of various cancers, including HCC.^[7–9]^ Within the context of tumors, IL-8 has been described to have dual pro-tumorigenic roles, including directly stimulating proliferation or transformation of tumor cells and recruiting a large number of immunosuppressive cells to the tumor.^[10]^ Recent clinical studies evaluating IL-8 levels in patients receiving immune checkpoint inhibition agents deduced that myeloid tumor infiltration driven by IL-8 contributes to resistance to immune checkpoint inhibition therapy.^[11–13]^ Targeting IL-8 or its receptors may provide a novel therapeutic strategy for tumor immune-based therapies.

Cytokines play various roles in tumorigenesis, tumor progression, and metastasis through cell-cell or cell-matrix interactions mediated by adhesion molecules.^[14]^ Integrins, a large family of cell-extracellular matrix adhesion molecules composed of α and β subunits, regulate whether cell survive, proliferate, and migrate in response to soluble growth factors and cytokines. Integrin expression varies greatly between normal and tumor tissues. It is becoming increasingly clear that tumor cells enhance the expression of integrins that promote their survival, proliferation, and migration.^[15]^ Among different integrins, integrin β3 serves a vital role in the progression and metastasis of various types of cancer, including HCC.^[16–19]^ Abnormal expression of integrin β3 is often associated with the development of HCC.^[19–22]^ Targeted therapies to integrin β3 have been reported in different types of cancer.^[23,24]^

Increasing evidence suggests that the close relationship between IL-8 and integrin β3 is critical for cancer progression.^[25,26]^ Our previous study has demonstrated that IL-8 promotes integrin β3 upregulation and cell invasion through PI3K/Akt pathway in HCC.^[22]^ However, the value of IL-8 and integrin β3 in prognosis prediction and targeted therapy of HCC remains unclear. In this study, we evaluated the relationship between IL-8 and integrin β3 expression in HCC. The association of IL-8 and integrin β3 expression levels with clinicopathological variables and patient outcome was investigated to determine whether IL-8 and integrin β3 might provide a theoretical basis for predicting the prognosis of patients with HCC after hepatectomy and for further studies on potential targeted therapeutic strategies.

## 2. Materials and methods

### 2.1. Patients and samples

This study enrolled 130 HCC patients who underwent hepatectomy in Shandong Provincial Hospital from 2013 to 2017 with a median follow-up of 53 months (range, 7–72 months). Clinical, laboratory, and histopathological data were retrospectively collected from the patients’ records. An invasion or tumor thrombus in the portal trunk or major branches of the portal vein was defined as macrovascular invasion. The inclusion criteria were as follows: definite pathological diagnosis, curative liver resection, and complete clinicopathological data. Patients who received preoperative anticancer treatment or had distant metastases were excluded. The study was conducted following the Declaration of Helsinki and approved by the Medical Ethics Committee of Shandong Provincial Hospital. Written informed consents were obtained from all patients.

### 2.2. Immunohistochemistry

After routine deparaffinization, rehydration, and antigen retrieval, the sections were incubated with anti-IL-8 antibody (1:2000, #ab18672, Abcam), or anti-integrin β3 antibody (1:100, #sc-52589, Santa Cruz) at 4°C overnight, followed by incubation with the corresponding secondary antibodies at 37°C for 30 minutes and visualization using diaminobenzidine. Each section was observed under a light microscope to evaluate the expression of IL-8 and integrin β3.

### 2.3. Evaluation of IL-8 and integrin β3 immunohistochemistry

IL-8 staining was mainly observed in the cytoplasm, while integrin β3 staining was visualized on the plasma membrane of tumor cells. Expression levels were evaluated based on the staining intensity and percentage of positively stained cells. The staining intensity was scored as 0 (no staining), 1 (weak staining), 2 (moderate staining), or 3 (strong staining). The percentage of stained cells was scored as 0 (no positive cells), 1 (≤5% positive cells), 2 (5%–50% positive cells), or 3 (≥50% positive cells). The sum of both parameters gave the final scores of each protein marker in each HCC sample, in which a final score <4 was defined as negative expression, and a score ≥4 was defined as positive expression.^[27]^

### 2.4. Statistical analysis

Statistical analyses were conducted using SPSS 22.0 software. The categorical variables were analyzed by Chi-square test. Survival analyses were performed using the Kaplan–Meier method and the log-rank test. A Spearman correlation was applied to evaluate the relationship between IL-8 and integrin β3 expression. Univariate and multivariate analyses were conducted with the Cox proportional hazard model to investigate those prognostic factors that predicted overall survival. *P* value < .05 was considered statistically significant.

## 3. Results

### 3.1. Clinicopathological information

A total of 130 patients (114 males and 16 females) undergoing surgical resection of hepatocellular carcinoma were enrolled in this study. Their median age was 56 years (range, 35–75 years old). The clinicopathological characteristics of all patients are listed in Table 1.

**Table 1:** Relationship between IL-8/integrin β3 expression and clinicopathological variables in patients with hepatocellular carcinoma.

Clinicopathological variables	n (%)	IL-8 expression			Integrin β3 expression		
		Positive (%)	Negative (%)	*P* value	Positive (%)	Negative (%)	*P* value
Gender				.316			.644
Male	114 (87.7)	56 (84.8)	58 (90.6)		50 (86.2)	64 (88.9)	
Female	16 (12.3)	10 (15.2)	6 (9.4)		8 (13.8)	8 (11.1)	
Age				.299			.086
≥55 yr	79 (60.8)	43 (65.2)	36 (56.3)		40 (69.0)	39 (54.2)	
<55 yr	51 (39.2)	23 (34.8)	28 (43.7)		18 (31.0)	33 (45.8)	
Macrovascular invasion				.047*			.084
Present	23 (17.7)	16 (24.2)	7 (10.9)		14 (24.1)	9 (12.5)	
Absent	107 (82.3)	50 (75.8)	57 (89.1)		44 (75.9)	63 (87.5)	
Tumor number				.069			.986
Multiple	38 (29.2)	24 (36.4)	14 (21.9)		17 (29.3)	21 (29.2)	
Solitary	92 (70.8)	42 (63.6)	50 (78.1)		41 (70.7)	51 (70.8)	
Maximal tumor size				.036*			.273
≥5 cm	67 (51.5)	40 (60.6)	27 (42.2)		33 (56.9)	34 (47.2)	
<5 cm	63 (48.5)	26 (39.4)	37 (57.8)		25 (43.1)	38 (52.8)	
Tumor differentiation				.008*			.513
I	5 (3.9)	0 (0)	5 (7.8)		1 (1.7)	4 (5.6)	
II	93 (71.5)	44 (66.7)	49 (76.6)		43 (74.2)	50 (69.4)	
III	32 (24.6)	22 (33.3)	10 (15.6)		14 (24.1)	18 (25)	
TNM stage				.001*			.045*
I–II	86 (66.2)	35 (53.0)	51 (79.7)		33 (56.9)	53 (73.6)	
III	44 (33.8)	31 (47.0)	13 (20.3)		25 (43.1)	19 (26.4)	
HBV infection				.899			.395
Yes	103 (79.2)	52 (78.8)	51 (79.7)		44 (75.9)	59 (81.9)	
No	27 (20.8)	14 (21.2)	13 (20.3)		14 (24.1)	13 (18.1)	
Serum AFP				.950			.496
≥200 ng/mL	43 (33.1)	22 (33.3)	21 (32.8)		21 (36.2)	22 (30.6)	
<200 ng/mL	87 (66.9)	44 (66.7)	43 (67.2)		37 (63.8)	50 (69.4)	
Hepatic cirrhosis				.964			.752
Yes	122 (93.8)	62 (93.9)	60 (93.8)		54 (93.1)	68 (94.4)	
No	8 (6.2)	4 (6.1)	4 (6.2)		4 (6.9)	4 (5.6)	

AFP = alpha-fetoprotein, HBV = hepatitis B virus, IL-8 = interleukin-8, TNM = tumor-node-metastasis.

* *P* value <.05.

### 3.2. Immunohistochemical analysis of IL-8 and integrin β3 expression in HCC

**Figure 1.**
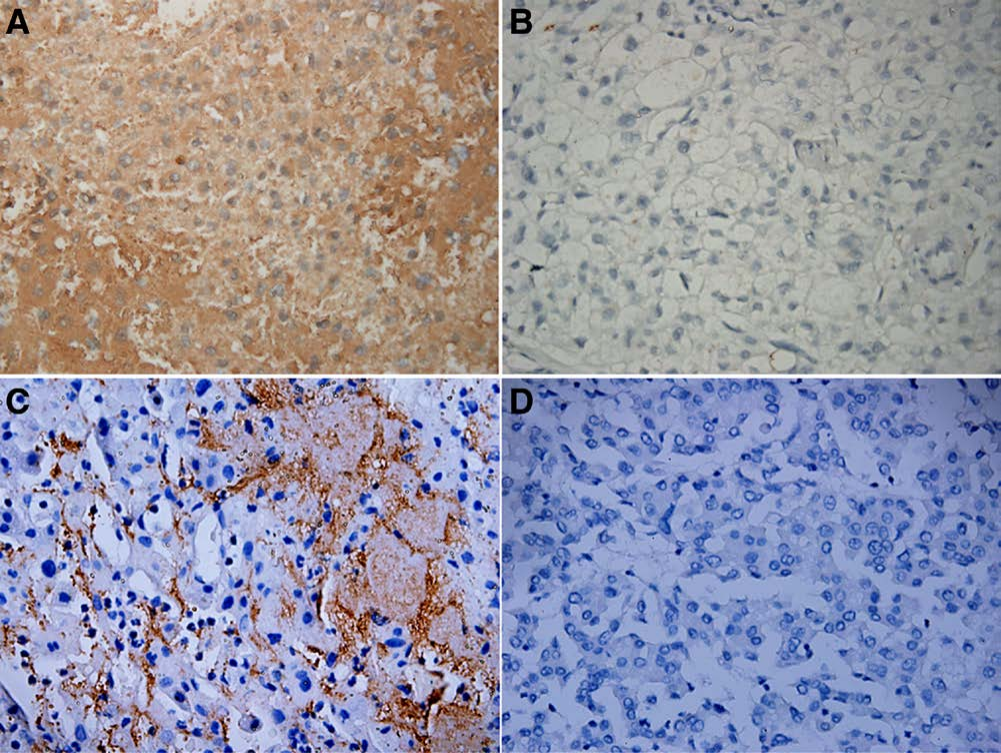
Immunohistochemical staining of IL-8 and integrin β3 in HCC (original magnification, ×400). (A) High IL-8 expression. (B) Low IL-8 expression. (C) Positive integrin β3 expression. (D) Negative integrin β3 expression. HCC = hepatocellular carcinoma, IL-8 = interleukin-8.

We detected the expression of IL-8 and integrin β3 in a cohort of 130 HCC patients using immunohistochemistry. IL-8 staining was observed mainly in the cytoplasm, while integrin β3 staining was detected on the plasma membrane of tumor cells (Fig. 1). Positive IL-8 expression was observed in 66 (50.8%) patients and positive integrin β3 expression was found in 58 (44.6%) patients (Fig. 1, Table 1).

### 3.3. Relationship between IL-8/integrin β3 expression and clinicopathological variables in HCC

The clinicopathological significance of IL-8/integrin β3 expression was evaluated by comparing the IL-8/integrin β3 positive group with the IL-8/integrin β3 negative group of patients with HCC (Table 1). The results revealed that IL-8 expression was highly associated with macrovascular invasion, tumor size, differentiation, and tumor-node-metastasis (TNM) stage of the tumors. IL-8 positive specimens exhibited a higher proportion of macrovascular invasion, larger tumor size, poor differentiation, and advanced TNM stage (*P* < .05, respectively; Table 1). There was a significant association between integrin β3 expression and TNM stage. Integrin β3 positive group exhibited a higher proportion of TNM III-staged tumors (*P* < .05, Table 1).

### 3.4. Association of relation between IL-8 and integrin β3 expression in HCC

Positive integrin β3 expression was observed in 54.5% of tissues positively expressing IL-8, while the percentage of tissues negatively expressing IL-8 was 34.4%. Integrin β3 expression had a positive association of relation with IL-8 expression based on the Spearman correlation analysis (*r* = 0.203, *P* = .021, Table 2).

**Table 2:** Association of relation between IL-8 expression and integrin β3 expression in hepatocellular carcinoma tissues.

IL-8 expression	Integrin β3 expression		Total
	Positive	Negative	
Positive	36	30	66
Negative	22	42	64
Total	58	72	130

IL-8 = interleukin-8.

### 3.5. Prognostic value of IL-8 and integrin β3 expression in patients with HCC after hepatectomy

**Figure 2.**
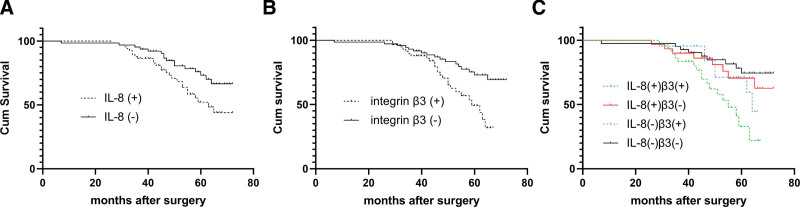
Survival curves of patients with HCC after hepatectomy based on the expression of IL-8 and integrin β3. (A) Patients with positive IL-8 expression had a significantly poorer overall survival rate than those with negative expression (*P* = .025; log-rank test: χ^2^ = 5.027). (B) Patients with positive integrin β3 expression had a significantly poorer overall survival rate than those with negative expression (*P* = .003; log-rank test: χ^2^ = 8.644). (C) Patients with positive IL-8 and integrin β3 expression had a significantly poorer overall survival rate than other groups (*P* = .003; log-rank test: χ^2^ = 13.737). HCC = hepatocellular carcinoma, IL-8 = interleukin-8.

Survival curves were generated via the Kaplan–Meier survival analysis. Patients with positive IL-8 expression (median survival time [MST] = 48 months) had a significantly poorer overall survival rate than those with negative expression (MST = 58 months; *P* = .025; log-rank test: *χ*^2^ = 5.027). Kaplan–Meier curves of overall survival based on IL-8 expression are illustrated in Figure 2A.

Patients with positive integrin β3 expression (MST = 48 months) had a significantly poorer overall survival rate than those with negative expression (MST = 57.5 months; *P* = .003; log-rank test: *χ*^2^ = 8.644). Kaplan–Meier curves of overall survival based on integrin β3 expression are presented in Figure 2B.

The 130 patients enrolled in this study were divided into 4 groups according to IL-8 and integrin β3 expression. The Kaplan–Meier analysis indicated that patients with positive IL-8 and integrin β3 expression (MST = 47 months) had a significantly poorer overall survival rate than other groups (*P* = .003; log-rank test: *χ*^2^ = 13.737). Kaplan–Meier curves of overall survival based on IL-8 and integrin β3 expression are shown in Figure 2C.

### 3.6. Univariate and multivariate analyses of prognostic factors related to survival of patients with HCC after hepatectomy

In order to investigate the prognostic value of IL-8 and integrin β3 expression in patients with hepatocellular carcinoma after hepatectomy, univariate and multivariate analyses were performed using the Cox proportional hazards regression model. Besides clinicopathological variables such as macrovascular invasion, tumor number, tumor size, TNM stage, and alpha-fetoprotein level, IL-8 and integrin β3 expression were proved to be able to predict prognosis based on univariate analysis (*P* < .05, respectively, Table 3). Variables with *P* < .05 were then selected as candidates for multivariate analysis. The results indicated that macrovascular invasion, advanced TNM stage, and integrin β3 expression were independent unfavorable prognostic factors (relative risk: 2.902, 3.277, and 2.001; *P* = .010, .034, and .046, respectively; Table 3).

**Table 3:** Univariate and multivariate analysis of association of clinicopathological variables with overall survival in patients with hepatocellular carcinoma after hepatectomy.

Variables	Univariate analysis			Multivariate analysis		
	Relative risk	95% CI	*P* value	Relative risk	95% CI	*P* value
Gender						
Male	1.000 (Ref.)					
Female	1.149	0.490–2.691	.749			
Age (yr)						
≥55	0.593	0.324–1.086	.090			
<55	1.000 (Ref.)					
Macrovascular invasion						
Present	10.744	5.249–22.004	<.001	2.902	1.284–6.560	.010
Absent	1.000 (Ref.)					
Tumor number						
Multiple	2.539	1.379–4.675	.003	1.452	0.754–2.797	.265
Solitary	1.000 (Ref.)					
Tumor size (cm)						
≥5	7.539	3.170–17.930	<.001	2.197	0.664–7.272	.198
<5	1.000 (Ref.)					
Differentiation						
I	0.000	0.000, >10^5^	.977			
II	0.615	0.323–1.170	.138			
III	1.000 (Ref.)					
TNM stage						
I–II	1.000 (Ref.)					
III	10.214	4.995–20.886	<.001	3.277	1.091–9.847	.034
HBV infection						
Yes	0.803	0.395–1.634	.545			
No	1.000 (Ref.)					
AFP (ng/mL)						
≥200	2.180	1.188–3.999	.012	1.156	0.603–2.215	.663
<200	1.000 (Ref.)					
Hepatic cirrhosis						
Yes	1.267	0.304–5.269	.745			
No	1.000 (Ref.)					
IL-8 expression						
Positive	2.126	1.137–3.974	.018	0.960	0.484–1.904	.907
Negative	1.000 (Ref.)					
Integrin β3 expression						
Positive	2.621	1.404–4.896	.002	2.001	1.011–3.959	.046
Negative	1.000 (Ref.)					

AFP = alpha-fetoprotein, CI = confidence interval, HBV = hepatitis B virus, IL-8 = interleukin-8, TNM = tumor-node-metastasis.

## 4. Discussion

Tumor progression relies on the ability of cancer cells to effectively invade surrounding tissues. Among the various mechanisms that contribute to tumor progression is the increase of the cancer cells’ motility and invasiveness influenced by a variety of soluble factors secreted from the surrounding microenvironment. Factors such as IL-8, transforming growth factor-β, and others are described as crucial molecules affecting various aspects of tumor progression.^[8]^ IL-8 has been shown to influence the biology, such as proliferation, migration, invasion, and angiogenesis of different types of cancer, including HCC.^[22,25,26,28]^ High circulating IL-8 levels are associated with increased tumor malignancy in HCC. Furthermore, serum IL-8 level is a significant and independent prognostic factor of survival in HCC.^[29,30]^ In tumor tissues, IL-8 expression is related to vascular invasion, tumor differentiation, and TNM stage. Moreover, high IL-8 expression also predicts poor postoperative prognosis in patients with HCC.^[31,32]^ In this study, we observed positive IL-8 expression in 50.8% of HCC tumor tissues. IL-8 positive specimens exhibited a higher proportion of macrovascular invasion, larger tumor size, poor differentiation, and advanced TNM stage. Patients with positive IL-8 expression had a significantly poorer overall survival rate than those with negative expression after hepatectomy.

As the main cell adhesion receptors for a variety of extracellular matrix components, the integrins, are a family of 24 transmembrane heterodimers comprising 18 α and 8 β integrin subunits that linked noncovalently. Upon binding to the extracellular matrix, the integrins organize the cytoskeleton and activate intracellular signaling, regulating complex cellular behaviors, including survival, proliferation, migration, differentiation, and various cell fate transition during inflammation and cancer. Altered integrin expression patterns have been linked to various types of cancer.^[15,33,34]^ Integrin β3 (β3, as part of αvβ3 and αIIbβ3 heterodimers) has been reported to be expressed in metastatic HCC cells and function as a modulator to promote metastatic phenotype of nonmetastatic HCC cells.^[19]^ Our previous study also demonstrated that integrin β3 was overexpressed in highly metastatic HCC cell lines compared with low metastatic cell lines.^[22]^ Previous findings suggest that high integrin β3 expression in anaplastic lymphoma kinase-rearranged non-small cell lung cancer is associated with tumor metastasis, more advanced tumor stages and a worse prognosis.^[35]^ Our results indicated that integrin β3 expression was significantly related to TNM stage in HCC. Patients with positive integrin β3 expression had a significantly poorer overall survival rate than those with negative expression and integrin β3 expression was an independent unfavorable prognostic factor in HCC after hepatectomy.

Investigating the association between IL-8 and integrin β3 expression may contribute to our understanding of the potential mechanisms underlying the tumor microenvironment involved in tumor progression. Our previous study has demonstrated that IL-8 promotes integrin β3 upregulation and the invasion of HCC cells through PI3K/Akt pathway.^[22]^ In this study, we found a positive relationship between IL-8 and integrin β3 expression in HCC, and patients with positive IL-8 and integrin β3 expression had a significantly shorter overall survival time.

In conclusion, our research indicated that increased expression of IL-8 and integrin β3 in HCC correlated with tumor progression and a poor survival. Integrin β3 expression was found to be an independent unfavorable prognostic factor in HCC after hepatectomy. Targeting integrin β3 might be a potential therapeutic approach in preventing tumor progression in HCC.

## Author contributions

**Funding acquisition:** Jiao Zhang, Mingze Ma, Fengkai Sun.

**Project administration:** Jiao Zhang.

**Writing—review & editing:** Jiao Zhang.

**Data curation:** Yi Yin.

**Resources:** Jiliang Tang.

**Software:** Jiliang Tang.

**Formal analysis:** Mingze Ma.

**Investigation:** Huimin Shen, Yingrong Zhang.

**Conceptualization:** Fengkai Sun.

**Supervision:** Fengkai Sun.

**Writing—original draft:** Fengkai Sun.
